# Evaluating the IgMi mouse as a novel tool to study B‐cell biology

**DOI:** 10.1002/eji.201847735

**Published:** 2018-10-26

**Authors:** Rinal Sahputra, Juan Carlos Yam‐Puc, Ari Waisman, Werner Muller, Kathryn J Else

**Affiliations:** ^1^ Lydia Becker Institute of Immunology and Inflammation Faculty of Biology, Medicine and Health Manchester Academic Health Science centre The University of Manchester Manchester United Kingdom of Great Britain and Northern Ireland; ^2^ Institute of Immunology and Immunotherapy University of Birmingham Birmingham United Kingdom of Great Britain and Northern Ireland; ^3^ Institute for Molecular Medicine University of Mainz Mainz Germany

## Abstract

The IgMi mouse fails to secrete antibodies or class switch its BCR from IgM. Our study reveals that other cellular compartments, including B‐cell subsets, DC subsets, GC B cells and T_FH_ cells are perturbed in the IgMi mouse, thus presenting important additional considerations when using the mouse to explore the role of secreted antibody.

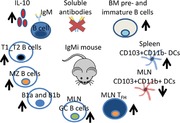

The current study aims to provide a detailed characterization of IgMi mice by comparing them to their wild type controls to evaluate the use of the IgMi mouse as a novel tool to study B‐cell biology. The IgMi mouse was firstly introduced in 2007 by Waisman et al., as IgH^μγ1/μγ1^ to address the importance of the cytoplasmic tail in B‐cell development 1, but subsequently became known as the IgMi mouse 2. The IgMi mouse was stated to have a normal B‐cell development, although its B cells only express IgM as a B‐cell receptor on the surface. Moreover, IgMi mice cannot produce any soluble antibodies as all constant regions in the IgH chain have been deleted 1. Thus, as a model to study B‐cell biology, the IgMi mouse offers great potential by virtue of its normal B‐cell development. However, there have been no subsequent publications providing a detailed description of the IgMi mouse under steady state, other than its inability to secrete antibodies.

We corroborate in the current study that IgMi mouse does not secrete any soluble antibodies. Total Ig analysis on the sera using ELISA showed that total Ig was not detected in the sera of IgMi mice with results below the background (Fig. 1A). Furthermore, no IgA antibody was detected in the stool of IgMi mice (Fig. 1B). However, the IgMi mouse showed enlarged secondary lymphoid organs (Fig. 1C) with elevations in B‐cell subsets, including B1a, B1b, transitional and marginal zone B cells (Fig. 1D–H). Natural antibodies, especially IgM, have been considered to play an important role in bridging the innate and adaptive immune systems. Thus, as shown in previous study using the AID^−/−^μS^−/−^ double mutant mouse 3, the current study revealed that secreted antibodies are important in cell extrinsic processes, controlling B‐cell subsets, such as B1 and MZ B cells. Interestingly, B‐cell development in the IgMi bone marrow was also altered as pre‐B cells and immature B cells were significantly higher, while mature B cells was significantly lower, compared to WT mice (Supplementary 1).

**Figure 1:**
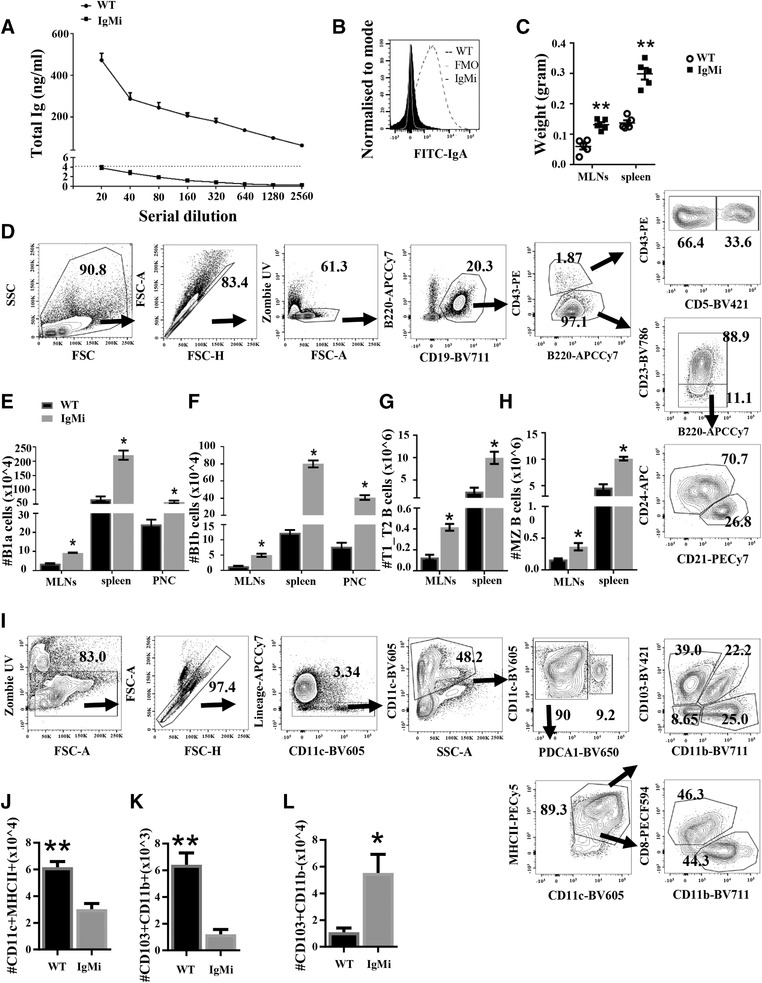
The absence of secreted antibodies in the IgMi mouse affects B cell and dendritic cell subsets. WT and IgMi mice were analyzed for (A) serial dilution of total Ig (ng/mL) using ELISA. Total Ig levels were quantified in serum samples of WT and IgMi mice relative to an IgA standard. (B) Faecal samples were stained with IgA using flow cytometry. (C) The comparison of weight of MLNs and spleen between IgMi mice and their age‐matched WT littermates. (D) Gating strategy to analyze B‐cell subsets in the spleen. B1a was defined as CD19^+^B220low/midCD43^+^CD5^+^, B1b as CD19^+^B220low/midCD43^+^CD5‐, marginal zone (MZ) as CD19^+^B220^+^CD5‐CD43‐CD23‐CD24‐CD21^+^ and transitional B cells as CD19^+^B220^+^CD5‐CD43‐CD23‐CD24^+^CD21^−^. (E–H) B‐cell subsets in IgMi mice were altered either in secondary lymphoid organs or peritoneal cavity (PNC). (I) Gating strategy for DC subsets. Two main DCs populations: conventional DCs (cDCs) and plasmacytoid DCs (pDCs). Conventional DCs was divided into migratory DCs and resident DCs. Migratory DCs consisted of four subpopulations: CD103^+^CD11b^−^, CD103^+^CD11b^+^, CD103‐CD11b^+^, and CD103‐CD11b^−^. Resident DCs was consisted of two subpopulations: CD8a^+^CD11b^−^ and CD8a‐CD11b^+^. Plasmacytoid DCs: CD11c^+^lineage‐PDCA‐1^+^. (J) Total cell number of CD11c^+^MHCII^+^ in MLNs. (K) Total cell number of CD103^+^CD11b^+^ in MLNs. (L) Total cell number of CD103^+^CD11b^−^ in the spleen. Sensitivity of the assay is shown as mean ± SD (dotted black line). (A&B) Data are representative of two separate experiments, *n* = 4/group, males, 12 weeks old. (C–L) Data are pooled from two separate experiments. All data are expressed as mean ± SEM. *p < 0.05, **p < 0.01 Mann–Whitney test.

B cells are also known to play a role in modulating the maturation and function of DCs 4, 5. Therefore, we wondered whether the lack of antibodies in IgMi mice would affect the subsets of DC in IgMi secondary lymphoid organs. Interestingly, IgMi mice had significantly lower MLN CD11c^+^MHCII^+^ DCs compared to WT littermates. Previous studies showed that the majority of CD11c^+^MHCII^+^ DC in the MLN under steady state are CD103^+^ DCs, including CD103^+^CD11b^+^ and CD103^+^CD11b^−^ 6. Interestingly, IgMi mice had significantly lower numbers of MLN CD103^+^CD11b^+^ DCs compared to WT littermates (Fig. 1I–K), while CD103^+^CD11b^−^ DCs were significantly increased in the spleen (Fig. 1L). Collectively, the data shows that lack of secreted antibody, directly or indirectly, affects B‐cell development in the bone marrow and changes the balance of B cell and DC subsets in secondary lymphoid organs. However, further studies are required to investigate the mechanisms behind those alterations in the IgMi mouse.

In contrast to the AID^−/−^μS^−/−^ double mutant mouse 3, IgMi mice only showed an increase in the relative percentage and number of germinal centre B cells (Fig. 2A–C), and number of GC in the MLNs (Fig. 2D–E). According to Zhang et al. 7, higher affinity antibodies secreted by B cells reenter the GC and negatively regulate GC formation by binding to follicular dendritic cells and limiting the access of B cells to antigens. Thus, in the absence of soluble antibodies, GC B cells in the IgMi mouse may be undergoing more proliferation and less apoptosis. Our study supports this hypothesis as GC B cells from IgMi mice showed increased proliferation and reduced apoptosis (Supplementary 2). We also investigated plasma cell and plasmablast populations in IgMi MLNs. Levels were very low under steady‐state conditions and we did not see any significant difference between genotype in any tissue (data not shown). In parallel to the increase in GC, the population of T‐follicular helper cells (T_FH_) in the MLNs of IgMi mice were also significantly increased (Fig. 2F–G). CD4^+^B220‐CXCR5^+^PD‐1^high^ cells are known as GC T_FH_ cells due to their ability to enter GCs and maintain stable interactions with B cells. Therefore, the increase in GC seen in IgMi mice correlates well with the increase in GC T_FH_. Interestingly, we did not see significant differences in CD4 and CD8 T cells in IgMi mice (data not shown).

**Figure 2:**
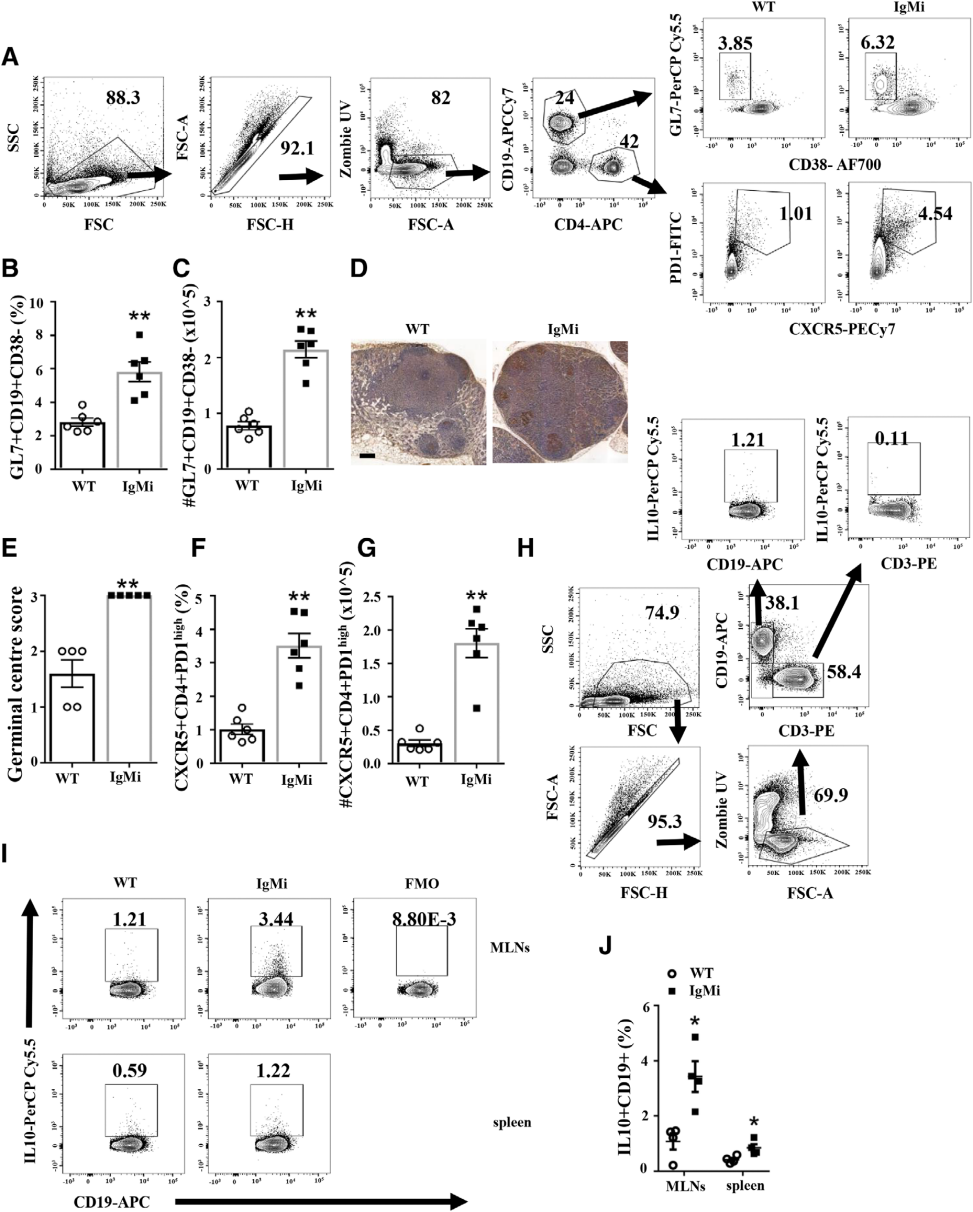
Germinal centers and T‐follicular helper cells are significantly increased in the MLNs of IgMi mice. WT and IgMi mice were analyzed by flow cytometry and histology (A) Gating strategy of GC B cells and T_FH_. GC were defined as CD19^+^GL7^+^CD38‐ and B220‐CD4^+^CXCR5^+^PD‐1high, respectively. (B&C) Relative percentage and total cell number of GCs. (D) Immunohistology staining of MLNs labeled with biotin peanut agglutinin (PNA) from WT and IgMi mice. Scale bar 500 μm. Images are representative from a single experiment with five mice per group. (E) The scores of germinal centers for PNA immunoreactivity based on the number of PNA^+^ within MLNs. (F&G) Relative percentage and total cell number of T_FH_. (H–J) MLN and spleen cells were restimulated with LPS in the presence of PMA, ionomycin and monensin (PIM) and IL‐10 was examined using flow cytometry. (H) Gating strategy of IL‐10 intracellular staining. (I) Representative data of IL‐10 producing B cells in MLNs and spleen. (J) Relative percentage of IL‐10 produced by B cells in MLNs and spleen. (A–G) Data are pooled from three separate experiments, *n* = 6, males, 12 weeks. (H–J) Data are pooled from two separate experiments, *n* = 4, males. All data are expressed as mean ± SEM. *p < 0.05, **p < 0.01 Mann–Whitney test.

B‐cell function is not only related to antibody production, but B cells are also able to produce cytokines including IL‐10 8. Due to its pleiotropic activities, IL‐10 is an important regulatory cytokine that is able to act both as an immunostimulator and immunosuppressor. A previous study showed that IL‐10 producing B cells were essential for GC development during malaria infection 9. Thus, the increase in GC cells seen in the IgMi mouse may reflect in part the increased ability by IgMi B cells to make IL‐10 (Fig. 2I–J). However, the mechanisms underlying the ability of IgMi B cells make more IL‐10 remains unknown. Further study is required to investigate whether this phenomenon is related to the lack of antibodies in IgMi mice, and also to address other unanswered questions in B cells biology, such as whether B cells secrete antibody and produce IL‐10 at the same time.

It remains controversial whether antibodies are important in regulating the composition of microbiota or not. Therefore, we also investigated if the lack of antibodies in IgMi mice would change the composition of the gut microbiota compared to their WT littermates. Surprisingly, we found that IgMi mice had a similar gut microbiota composition to their WT littermates based on microbiome analysis using DGGE and real‐time PCR (Supplementary 3). It is possible that, the insignificant difference in gut microbiota between the IgMi mice and WT littermates could be related to maternal immunity 10, however, although both genotypes were breast‐fed by a heterozygous mother, flow cytometric analyses of faecal IgA failed to reveal the presence of any antibody in the IgMi mouse (Fig. 1B). Furthermore, in keeping with the absence of any differences in the gut microbiota in IgMi and WT mice, there were no significant differences in expression levels of IFN‐γ and IL‐17 in the gut in IgMi mice compared to WT littermates (Supplementary 4).

In conclusion, the IgMi mouse represents a powerful model system which, in combination with other B‐cell transgenic mice, can be used to investigate the biology of B cells. However, it is important to be aware that steady‐state differences beyond lack of antibodies exist including differences in B‐cell subsets, B‐cell propensity to make IL‐10, dendritic cell subsets, germinal centers, and T‐follicular helper cells.

## Conflict of interest

The authors declare no commercial or financial conflict of interest.

## Supporting information

Supplementary Figure 1. B cell development in bone marrow (BM). (A) Gating strategy to analyse B cell sub‐populations in the BM. Pro B cells were defined as B220+IgM‐CD25‐CD43+CD19+c‐Kit+, pre B cells as B220+IgM‐CD25+CD43‐, immature B cells as B220lowIgM+AA4.1+ and mature B cells as B220highIgM+AA4.1‐. (B‐I) Relative % and total cell number of B cell subsets in BM. Pre B cells and immature B cells are higher in frequencies and absolute numbers whereas mature B cells are lower in IgMi mice. All data are expressed as mean ± SEM. *P<0.05, **P<0.01 Mann‐Whitney test.
Supplementary Figure 2. Proliferation and apoptosis in GCs from MLNs. (A) Gating strategy to analyse proliferation and apoptosis in GC B cells from MLNs. GC B cells were defined as CD138‐B220+CD38‐/lowFas+. Proliferating Ki67+ cells and apoptotic active caspase‐3+ cells were gated on GC B cells. (B) Relative % of GC B cells. (C&D) Relative % of Ki67+ cells and apoptotic active caspase‐3+ cells , respectively. Higher GCs frequencies are shown with higher proliferation and lower apoptosis in IgMi mice. All data are expressed as mean ± SEM. *P<0.05, **P<0.01 Mann‐Whitney test.
Supplementary Figure 3. The intestinal microbiota of WT and IgMi mice do not differ. Faecal samples were collected 4 weeks post weaning. Microbiota diversity (A&B) and bacterial genera (C‐H), including Bacteriodes, segmented filamentous bacteria (SFB), total Helicobacter, Lactobacillus, Enterobacter, and Verrucomicrobiales using DGGE and real time qPCR, respectively. (A) Nonmetric multidimensional scaling (nMDS) were calculated using R software comparing species distribution between WT (dark round shape) and IgMi (white round shape) in individual mice. Axis represents the scale of Euclidean distance between the samples. (B) Shannon index to analyse species abundance. qPCR data were normalised using 16 S and fold changes are shown relative to WT naive. (A&B) Data are representative of 2 separate experiments. (C‐H) Data are pooled from 2 separate experiments, n=12, males, 8 weeks old. All data are expressed as mean ± SEM
Supplementary Figure 4. Pro‐inflammatory cytokines in the guts of WT and IgMi mice. IFN‐γ and IL‐17 expressions in the colon of naive IgMi and WT (A&B). The expression of genes was normalised using eef. Fold changes are shown relative to WT naive. Data are pooled from 2 separate experiments, n=8, males, 12 weeks old. All data are expressed as mean ± SEM

Peer review correspondence

Supporting Information
