# Supplementary material for: Evaluating the IgMi mouse as a novel tool to study B‐cell biology
Source: Eur J Immunol. 2018 Oct 26;48(12):2068–71. doi: 10.1002/eji.201847735 (PMC6750126; doi:10.1002/eji.201847735)
Supplement: Supplementary file 1 — Supplementary Figure 1. B cell development in bone marrow (BM). (A) Gating strategy to analyse B cell sub‐populations in the BM. Pro B cells were defined as B220+IgM‐CD25‐CD43+CD19+c‐Kit+, pre B cells as B220+IgM‐CD25+CD43‐, immature B cells as B220lowIgM+AA4.1+ and mature B cells as B220highIgM+AA4.1‐. (B‐I) Relative % and total cell number of B cell subsets in BM. Pre B cells and immature B cells are higher in frequencies and absolute numbers whereas mature B cells are lower in IgMi mice. All data are expressed as mean ± SEM. *P<0.05, **P<0.01 Mann‐Whitney test. Supplementary Figure 2. Proliferation and apoptosis in GCs from MLNs. (A) Gating strategy to analyse proliferation and apoptosis in GC B cells from MLNs. GC B cells were defined as CD138‐B220+CD38‐/lowFas+. Proliferating Ki67+ cells and apoptotic active caspase‐3+ cells were gated on GC B cells. (B) Relative % of GC B cells. (C&D) Relative % of Ki67+ cells and apoptotic active caspase‐3+ cells , respectively. Higher GCs frequencies are shown with higher proliferation and lower apoptosis in IgMi mice. All data are expressed as mean ± SEM. *P<0.05, **P<0.01 Mann‐Whitney test. Supplementary Figure 3. The intestinal microbiota of WT and IgMi mice do not differ. Faecal samples were collected 4 weeks post weaning. Microbiota diversity (A&B) and bacterial genera (C‐H), including Bacteriodes, segmented filamentous bacteria (SFB), total Helicobacter, Lactobacillus, Enterobacter, and Verrucomicrobiales using DGGE and real time qPCR, respectively. (A) Nonmetric multidimensional scaling (nMDS) were calculated using R software comparing species distribution between WT (dark round shape) and IgMi (white round shape) in individual mice. Axis represents the scale of Euclidean distance between the samples. (B) Shannon index to analyse species abundance. qPCR data were normalised using 16 S and fold changes are shown relative to WT naive. (A&B) Data are representative of 2 separate experiments. (C‐H) Data are pooled from 2 [file EJI-48-2068-s001.pdf]

European Journal of Immunology

Supporting Information
for
DOI 10.1002/eji.201847735

Rinal Sahputra, Juan Carlos Yam-Puc, Ari Waisman, Werner Muller and Kathryn J Else

Evaluating the IgMi mouse as a novel tool to study B-cell biology

© 2018 WILEY-VCH Verlag GmbH & Co. KGaA, Weinheim

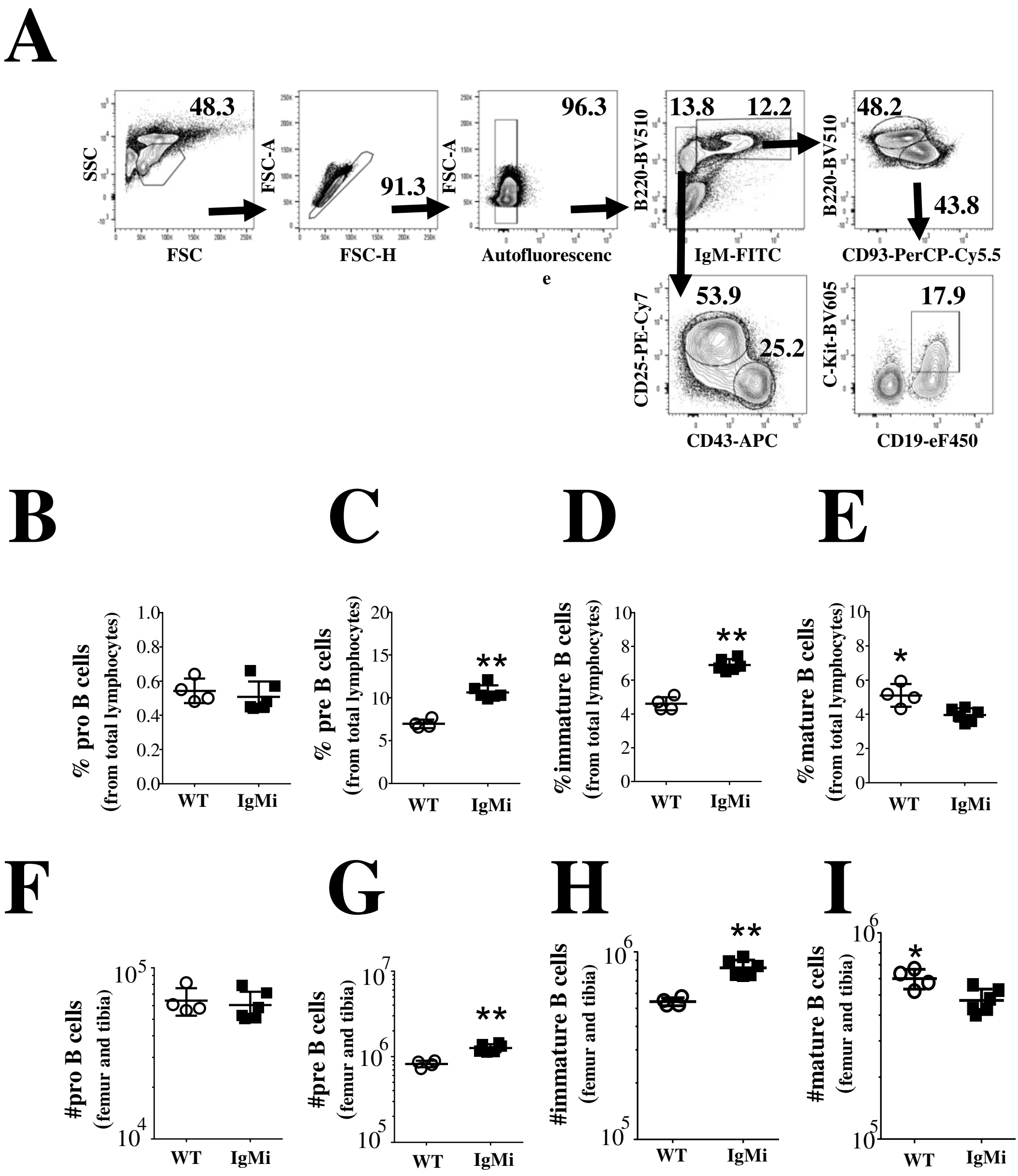

Supplementary Figure 1. B cell development in bone marrow (BM). (A) Gating strategy to analyse B cell sub-populations in the BM. Pro B cells were defined as B220+IgM-CD25-CD43+CD19+c-Kit+, pre B cells as B220+IgM-CD25+CD43-, immature B cells as B220lowIgM+AA4.1+ and mature B cells as B220highIgM+AA4.1-. (B-I) Relative % and total cell number of B cell subsets in BM. Pre B cells and immature B cells are higher in frequencies and absolute numbers whereas mature B cells are lower in IgMi mice. All data are expressed as mean ± SEM. *P<0.05, **P<0.01 Mann-Whitney test.

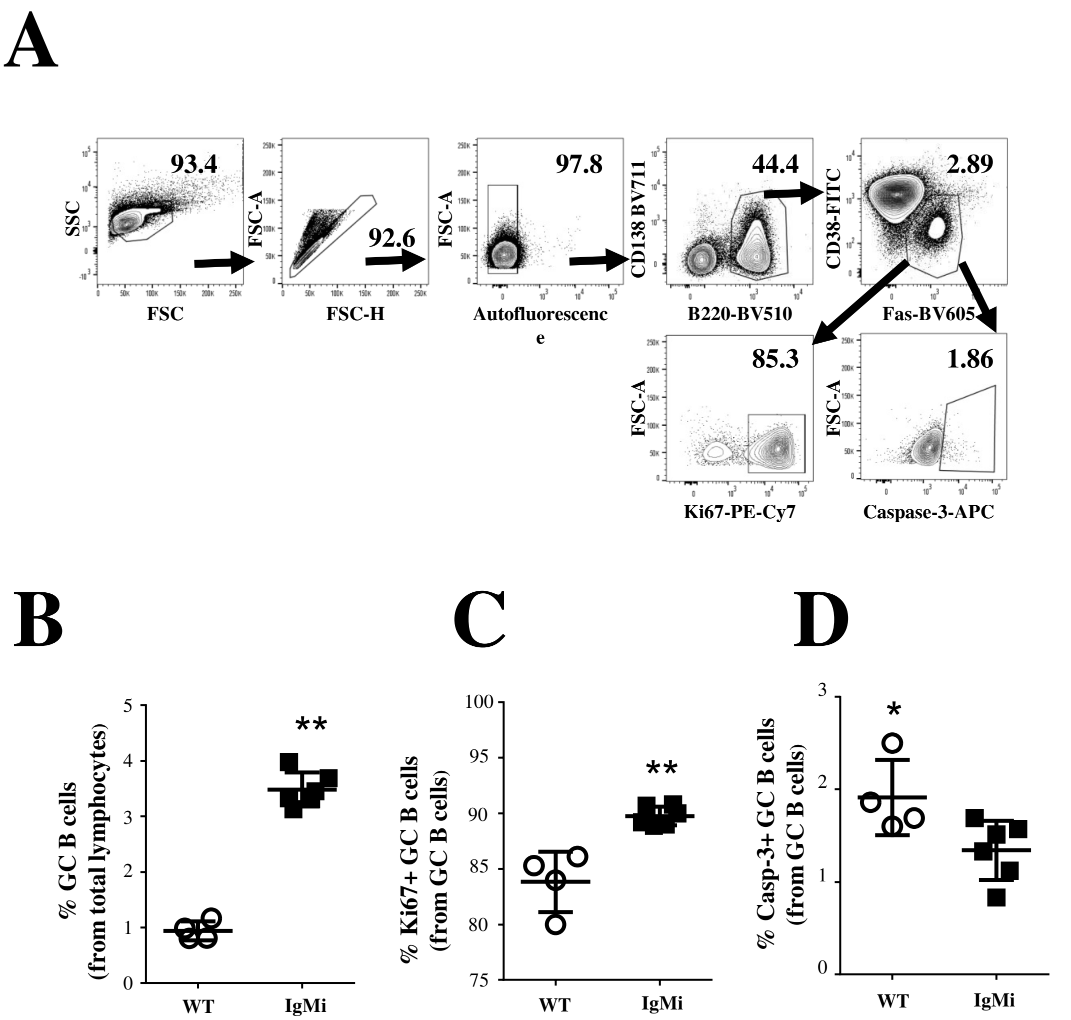

Supplementary Figure 2. Proliferation and apoptosis in GCs from MLNs. (A) Gating strategy to analyse proliferation and apoptosis in GC B cells from MLNs. GC B cells were defined as CD138-B220+CD38-/lowFas+. Proliferating Ki67+ cells and apoptotic active caspase-3+ cells were gated on GC B cells. (B) Relative % of GC B cells. (C&D) Relative % of Ki67+ cells and apoptotic active caspase-3+ cells , respectively. Higher GCs frequencies are shown with higher proliferation and lower apoptosis in IgMi mice. All data are expressed as mean ± SEM. *P<0.05, **P<0.01 Mann-Whitney test.

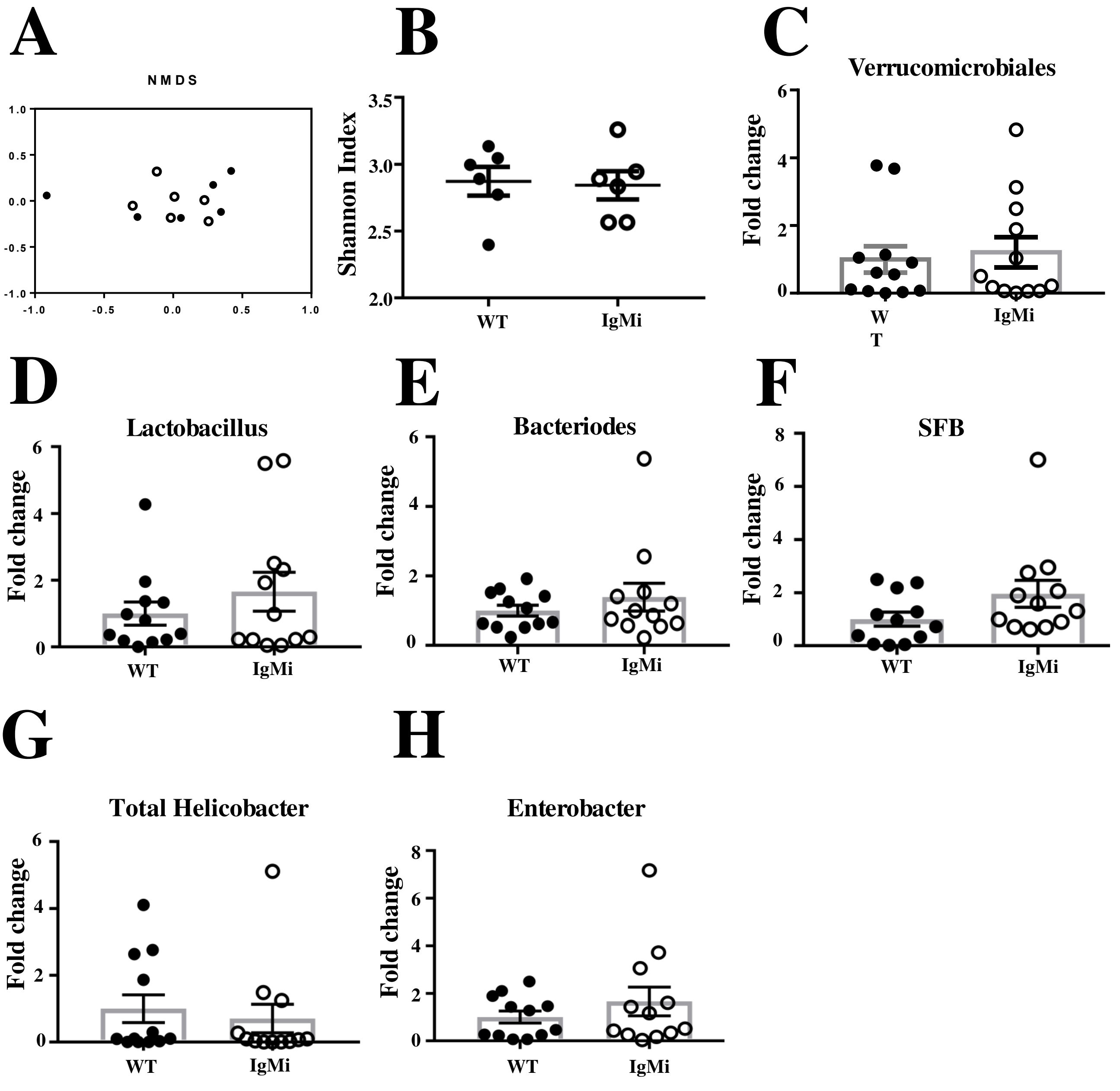

Supplementary Figure 3. The intestinal microbiota of WT and IgMi mice do not differ. Faecal samples were collected 4 weeks post weaning. Microbiota diversity (A&B) and bacterial genera (C-H), including Bacteriodes, segmented filamentous bacteria (SFB), total Helicobacter, Lactobacillus, Enterobacter, and Verrucomicrobiales using DGGE and real time qPCR, respectively. (A) Nonmetric multidimensional scaling (nMDS) were calculated using R software comparing species distribution between WT (dark round shape) and IgMi (white round shape) in individual mice. Axis represents the scale of Euclidean distance between the samples. (B) Shannon index to analyse species abundance. qPCR data were normalised using 16 S and fold changes are shown relative to WT naive. (A&B) Data are representative of 2 separate experiments. (C-H) Data are pooled from 2 separate experiments, n=12, males, 8 weeks old. All data are expressed as mean ± SEM

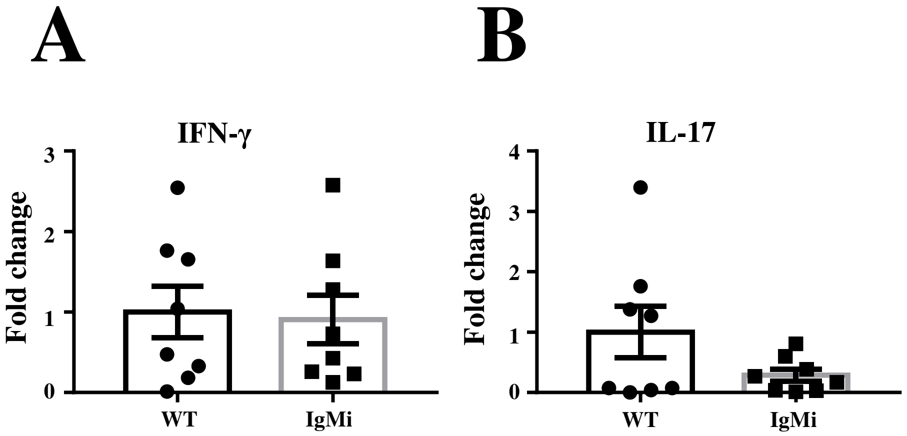

Supplementary Figure 4. Pro-inflammatory cytokines in the guts of WT and IgMi mice. IFN-γ and IL-17 expressions in the colon of naive IgMi and WT (A&B). The expression of genes was normalised using eef. Fold changes are shown relative to WT naive. Data are pooled from 2 separate experiments, n=8, males, 12 weeks old. All data are expressed as mean ± SEM
