# Supplementary material for: Evaluating the IgMi mouse as a novel tool to study B‐cell biology
Source: Eur J Immunol. 2018 Oct 26;48(12):2068–71. doi: 10.1002/eji.201847735 (PMC6750126; doi:10.1002/eji.201847735)
Supplement: Supplementary file 3 — Supporting Information [file EJI-48-2068-s003.docx]

**Material and Methods**

**Animals**

The IgMi knockout colony was maintained using breeding pairs of specific-pathogen-free male and female heterozygous (Het) IgMi mice on a C57BL/6 background. The resulting wild type (WT), knock out (KO) IgMi, and Het offspring were maintained in individually ventilated cages (IVC) in the animal facility at the University of Manchester under specific pathogen free conditions. 8-12 weeks IgMi and WT littermates were used for the study and all procedures carried out on animals were performed under the regulation of the Home Office Scientific Procedures Act (1986).

**Genotyping**

The genotype of the IgMi mice was assessed by PCR. DNA from ear punches was extracted using a tissue PCR kit (Sigma Aldrich). 100 µL of extraction and 25 µL of tissue prep solution were added to each ear punch before samples were heated at 55^0^C and 95^0^C for 10 minutes and 3 minutes respectively. 100 µL of neutralization solution was added, vortexed, and 200 µL resulting solution transferred to a fresh tube. The amplification reaction consisted of 1 µL of DNA extract, 5 µL RedTaq (Sigma Aldrich), 3.76 µL nuclease free water, 0.12 µL primer 1 ((Luz6-Eurofins) CCTTCCTCCTACCCTACAAGCC) and 0.12 µL primer 2 ((Luz8-Eurofins) GAGACGAGGGGGAAGACATTTG) in 10 µL of total volume. The specific bands for KO IgMi, WT and Het were identified on Benchtop UV transluminator.

**Total Immunoglobulin (Ig)**

The total immunoglobulin in sera was measured as previously (48) with a slight modification. Briefly, an ELISA plate (BrandTech Scientific, Inc) was coated with 2 µg/ml of polyclonal goat anti mouse Ig (BD Biosciences) in a 0.05M carbonate bicarbonate buffer of pH 9.6 overnight. The plate was blocked with 3% bovine serum albumin (BSA) for 45 minutes at 37^0^C. A mouse IgA standard (Southern Biotech) was used to quantify total immunoglobulin, and was double diluted 16 times from an initial concentration of 500 ng/ml. Serum samples were double diluted eight times starting at 1/200. Serum immunoglobulin was detected using a horse radish peroxidase (HRP)-conjugated rat anti mouse Ig, κ light chain (BD Biosciences). The plate was developed with TMB substrate as previously described. Total Ig concentration in the sera was calculated using the standard curve.

**Immunohistochemistry staining**

Mesenteric lymph nodes were placed in 10% neutral buffered formalin (NBF) for 24 hours and stored in 70% ethanol. Tissues were paraffin embedded and 5μm thick sections were cut using the HM325 MICROM microtome (MICROM). Sections were mounted onto slides. Germinal centre staining was performed using biotin labelled PNA (Vector laboratories, Burlingame, CA) and a semi-quantitative scoring scale was used as previously described (49).

**Stool samples collection, DNA extraction and microbiome analysis**

Microbiomes from stool were analysed using a method previously described (50). Briefly, fresh stools were collected from individual mice 4 weeks after weaning. Samples were stored at -80^0^C until DNA extraction was performed. DNA was extracted using the QIAamp DNA stool mini kit (Qiagen) according to the manufacturer instructions. Gut microbiomes were characterised with PCR by amplifying the V3 region of the 16S rRNA gene, using the 341F- CGCCCGCCGCGCGCGGCGGGCGGG and 518R-ATTACCGCGGCTGCTGG primers. PCR products were purified (QiaGEN Minelute kit) before loading onto a denaturing gradient gel electrophoresis (DGGE) gel. The gel was imaged using a bench top UV transilluminator, BioDoc-It Imaging System (UVP) and analysed using Phoretix 1D software for the presence or absence of the bands. Non-parametric multidimensional scaling (NMDS) analysis and Shannon diversity analysis were performed using R studio software.

**16S rRNA analyses**

Real time PCRs were performed in 96 well plates (Starlab) in Applied Biosystems® 7500 Fast Real-Time PCR Systems (Life Technologies). A Brilliant III Ultra-Fast SYBR® Green QPCR Master Mix (Agilent Technologies) was used to determine the relative expression of bacterial genera, including *Verrucomicrobiales*, *Lactobacillus*, *Bacteriodes*, segmented filamentous bacteria (SFB), total *Helicobacter* and *Enterobacter*. 1 µl (100 ng/ µl) amplified DNA was mixed with the following: 5 µl SYBR Green PCR master mix, 0.5 µl of forward and reverse primers each taxa, 0.15 µl of diluted reference dye and nuclease-free water in a total volume of 10 µl. All of the 16S rRNA gene-targeted group-specific primers used in this study were as previously described (51). The primer sequences for *Lactobacillus*, *Bacteriodes*, segmented filamentous bacteria (SFB), total *Helicobacter* and *Enterobacter* were purchased from Invitrogen, whilst the primer for *Verrucomicrobiales* was from Eurofins Biogenomic

**qPCR**

The production of IFN-γ and IL-17 mRNA in the caecal mucosa was examined using RT-PCR. The total RNA was extracted from caecal tips using TRIzol reagent (Invitrogen) and stored at -80°C prior to use. cDNA was synthesised from mRNA using the High-Capacity cDNA Reverse Transcription Kits (Life Technologies) containing recombinant moloney murine leukaemia virus reverse transcriptase (MultiScribe™ RT, Life Technologies). After cDNA synthesis, a Brilliant III Ultra-Fast SYBR® Green QPCR Master Mix (Agilent Technologies) was used as described above. The primer sequences used were IFN-γ: 5-TGAGCTCATTGAATGCTTGG-3 and 5-ACAGCAAGGCGAAAAAGGAT-3; IL-17: 5-TGAGCTTCCCAGATCACAGA-3 and 5-TCCAGAAGGCCCTCAGACTA-3; eef: 5-TGTCAGTCATCGCCCATGTG-3 and 5-CATCCTTGCGAGTGTCAGTGA-3. All primers were purchased from Eurofins Biogenomic. Details of the PCR program used are shown in Supplemetary Table 1)

**Flow cytometry**

Cell surface markers

Single-cell suspensions from BM, MLN and spleen cells were stained for cell surface markers. MLN and spleen cells were also stained for intracellular markers. Samples were stained for live dead (Zombie UV, Biolegend) and Fc block (eBiosciences) prior to cell surface/intra cellular markers staining. Samples were read on a BD LSR Fortessa flow cytometer (BD Biosciences) and data was analysed using FlowJo X (Tree Star, Inc).

Cell surface markers: anti-B220 (RA3-6B2); anti-CD19 (6D5); anti-CD3 (17A2); anti-CD43 (S11); anti-CD5 (53-7.3); anti-CD21 (7E9); anti-CD24 (30-F1); anti-IgD (11-26C.2a);anti-CD93 (AA4.1); anti-Ly6C (HK1.4); anti-CD103 (2E7); anti-CD11c (N418); anti-CD317/PDCA-1 (927); anti CD11b (M1/70); anti-CD64 (X54-5/7.1); anti-C-Kit (ACK2); anti-I-A/I-E (M5/114.15.2); anti MU/HU GL7 (GL7); anti-CD38 (90); anti-CD279 (PD-1) (29F.1A12); anti-CD185/CXCR5 (L138D7); anti-CD138 (281-2) purchased from Biolegend. Anti-CD23 (B3B4); anti-CD8a (53-6.7); anti-Fas (Jo2) purchased from BD Biosciences. Anti-CD25 (PC61.5); anti-CD45 (30-F11); anti-ly6G (RB6-8C5); anti-NK.1 (PK136); anti-Ter119 (Ter-119) purchased from eBiosciences. Anti-IgM (polyclonal) purchased from Southern Biotech.

Intracellular analysis

Analysis of IL-10 production was as described previously (52) with a slight modification. Briefly, isolated mesenteric lymph nodes lymphocytes and spleenocytes were resuspended (1 × 10^6^ cells/ml) in complete RMPI 1640 medium (10% FCS, 5ml L-Glut, 1x pen-strep and 5 × 10−5 M 2-mercaptoethanol (all from Gibco, Carlsbad, CA)) with LPS (10 μg/ml, *Escherichia coli* serotype 0111: B4, Sigma), PMA (50 ng/ml; Sigma), ionomycin (500 ng/ml; Sigma), and monensin (2 μM; eBioscience) for 5 hours, in 24-well flat-bottom plates. Anti-Mouse CD16/CD32 Fc Block (eBioscience) and live dead (Zombie UV, Biolegend) were added before cell surface staining. Stained cells were fixed and permeabilized using a Cytofix/Cytoperm kit (BD PharMingen) according to the manufacturer’s instructions and stained with anti-IL-10 (JES5-16E3) or isotype control Rat IgG2b (RT K4530) purchased from Biolegend mAb.

To detect Ki67+ cells and apoptosis active Casp-3+ cells, MLN cells were stained for cell surface and intracellular markers. After cell surface markers staining, cells were then fixed and permeabilised using the Foxp3/Transcription Factor Staining Buffer Set (eBioscience) for 60 minutes at 4°C. Samples were washed using 1 x Permeabilisation Buffer and blocked for non-specific binding by incubating samples in 50μl Anti-Mouse CD16/CD32 Fc Block (eBioscience) for 15 minutes at room temperature. Without washing, 50μl conjugated antibody for detection of intracellular antigen(s) was added to samples and incubated at room temperature for 30 minutes. Samples were then washed with Permeabilisation Buffer and resuspended in 200μl FACS buffer (0.05% azide, 0.1% BSA). Samples were read on a BD LSRFortessa flow cytometer (BD Biosciences) and data was analysed using FlowJo X (Tree Star, Inc). anti-Ki67 (B56) and anti-Casp3 (C92-605) were purchased from BD Pharmingen.

**Statistics**

Statistical analysis was performed using Prism4 (Graph-Pad software Inc., La Jolla, CA). The significant differences between two groups (P<0.05) were analysed with the Mann-Whitney test.
