# Supplementary material for: Evaluating the IgMi mouse as a novel tool to study B‐cell biology
Source: Eur J Immunol. 2018 Oct 26;48(12):2068–71. doi: 10.1002/eji.201847735 (PMC6750126; doi:10.1002/eji.201847735)
Supplement: Supplementary file 2 — Peer review correspondence [file EJI-48-2068-s002.pdf]

European Journal of Immunology

Evaluating the IgMi mouse as a novel tool to study B cell biology

Rinal Sahputra, Juan Carlos Yam-Puc, Ari Waisman, Werner Muller and Kathryn J Else

Correspondence: Dr. Kathryn Else, Lydia Becker Institute of Immunology and Inflammation, Faculty of Biology, Medicine and Health, The University of Manchester, Manchester Academic Health Science centre, Manchester, United Kingdom

---

Review Timeline:	Submission date:	04-Jun-2018
	First Editorial decision:	13-Jul-2018
	Revision received:	05-Sep-2018
	Accepted:	05-Oct-2018

Handling Executive Committee member: Prof. Jürgen Wienands

Please note that the correspondence below does not include the standard editorial instructions regarding preparation and submission of revised manuscripts, only the scientific revisions requested and addressed.

First Editorial Decision
13-Jul-2018

Dear Dr. Else,

Manuscript ID eji.201847735 entitled "Evaluating the IgMi mouse as a novel tool to study B cell biology" which you submitted to the European Journal of Immunology has been reviewed. The comments of the referees are included at the bottom of this letter. We are sorry for the delay in the peer review, but one of the referees was quite delayed with his report so we had to seek help of another referee.

Please note that Executive Committee is requesting that you convert your manuscript to a Letter to the Editor, focus on the major findings and use referees™ comments as a guideline to improve the study.

In more details, we think that you should modify the manuscript so that:

Fig 1 “ is moved to Supporting Information

Fig 2 and 3 are merged into one figure containing the key information about B cells and DCs. The rest should be shifted to Supporting Information

Fig. 4 and 5 are merged into one Figure

Fig. 6 and 7 are moved to Supporting Information

A revised version of your manuscript that takes into account the comments of the referees will be reconsidered for publication. Should you disagree with any of the referees™ concerns, you should address this in your point-by-point response and provide solid scientific reasons for why you will not make the requested changes.

You should also pay close attention to the editorial comments included below. **In particular, please edit your figure legends to follow Journal standards as outlined in the editorial comments. Please show fluorochrome axis labels and scaling in flow cytometry plots. Failure to do this will result in delays in the re-review process.**

Please note that submitting a revision of your manuscript does not guarantee eventual acceptance, and that your revision will be re-reviewed by the referees before a decision is rendered.

If the revision of the paper is expected to take more than three months, please inform the editorial office. Revisions taking longer than six months may be assessed by new referees to ensure the relevance and timeliness of the data.

Once again, thank you for submitting your manuscript to European Journal of Immunology and we look forward to receiving your revision.

Yours sincerely,
Nadja Bakocevic

On behalf of
Prof. Jürgen Wienands

Dr. Nadja Bakocevic
Editorial Office
European Journal of Immunology
e-mail: ejied@wiley.com
www.eji-journal.eu

European Journal of Immunology

****

Reviewer: 1

Comments to the Author

In their manuscript "Evaluating the IgMi mouse as a novel tool to study B cell biology" , Sahputra et al., describe a mouse model with B cells expressing IgM on the surface which cannot secret antibodies. The IgMi model can be used to study the effects of a lack of soluble antibodies on B cell homeostasis and on other cell types such as DCs or T cell subsets.

Therefore, this study is of potential interest to clarify the regulatory role of soluble antibodies and the regulatory capacity of B cells.

Comments:

The abstract does not really provide all essential information about the IgMi mouse model. Not everyone is familiar with this model. Please explain the relevance of this mouse model in more detail.

In their manuscript the authors use a mouse model named IgMi and refer to the following reference: Waisman et al., 2007. In Waisman et al., 2007, however, the authors use different nomenclatures for their mouse strains which makes it quite difficult to figure out which mouse they used in the present study. Please be consistent in the description and nomenclature of this mouse line.
I recommend to include a schematic drawing of the genetic features and alterations of the IgMi mouse.

The authors found enlarged spleens and MLNs in the IgMi mouse. What about transitional B cells. Are they changed? Is B cell development in the bone marrow completely normal?

In Figure 2 the authors present data that show an increase in B1 cells and MZB cells. Are the cell numbers normalized to the increased number of total splenocytes? What about CD4 and CD8 T cells in the Spleen (and the MLN)?
Is there more output from the bone marrow?
Did the authors analyze the B1 compartment in the peritoneum as well?

In Figure 4 the authors nicely show an increase in GL7+ GC B cells.
Are IgMi B cells "hyperactive" in general? Is there in vitro data available (LPS Stimulation, anti CD40/IL4 etc..) regarding proliferation, GL7 upregulation, apoptosis? Please clarify this point.

Based on the finding that IgMi mice harbor more GL7+ GC cells:
What about plasmablasts and plasma cells. Are they generated in this mouse? Is there an enrichment of IgM+ plasmablasts?

Statistics:
Please clearly state the total number of mice used for every experiment. Sometimes it is not clear; Figure 2: "Data are pooled from two experiments, n=4". Is that n=8 in total? Or was it two times 2 animals resulting in n=4. Please clarify.
For n=4 a t-test is not appropriate because of its low statistical power for small n. Please use other appropriate statistical tests.

Reviewer: 2

Comments to the Author
In this study, Sahputra et al. report the characterization of cell subsets in IgMi mice that cannot secrete antibodies compared to controls. They find alteration in B cell subsets, DC subsets, and TFH cells. Although this is possibly of interest for people who are considering working with these mice, these descriptive results do not reach the level of significance necessary for publication in the European Journal of Immunology. Part of these findings are already known eg B cell subsets abnormalities in the absence of secreted Abs (muS mice or Aid-/-muS mice).

Comments: Supplementary Figure 3 is missing in the figure files (cited in line 148).

Reviewer: 3

Comments to the Author
Overall this is a very useful study. Personally, I have wondered why more information on the IgMi mouse was not available, so this is a welcome addition to the literature.

There is always more to do, therefore I will not ask for additional experimentation. I have only minor comments.

It would be helpful for the authors to emphasize the degree and timing with which WT and IgMi/i mice were housed together. This is particulary important for the microbiota data in Figure 6, but may also be relevant to several other figures. Moreover, the authors should consider emphasizing the caution with

which the microbiota data are discussed. The microbiota literature is becoming clogged with conflicting reports, and at least some of this reflects different approaches to housing and co-housing genotype-disparate mice. Really drilling down on these issues can be quite involved (see PMID 28614717, for example).

Note: typo " Figure 1C is actually 1B

First Revision – authors' response
05-Sep-2018

Responses to Reviewers

Throughout this response letter, the comments of reviewers are in normal text and underlined and our responses are italicised, and indicated by referring to text lines where the amends appear in our revised manuscript

Executive Committee:

Please convert your manuscript to a Letter to the Editor, focus on the major findings and use referees' comments as a guideline to improve the study. We have now converted our manuscript to a Letter to the editor style

Fig 1 – is moved to Supporting Information. We have converted this data into a small Figure (1C) as we feel the data is an important part of our opening figure.

Fig 2 and 3 are merged into one figure containing the key information about B cells and DCs. The rest should be shifted to Supporting Information. The key information in Fig 2 and 3 now appear as Fig 1 with the other data in supporting information

Fig. 4 and 5 are merged into one Figure. Fig 4 and 5 now appear as Fig 2

Fig. 6 and 7 are moved to Supporting Information. Fig 6 and 7 are now in Supporting information

In particular, please edit your figure legends to follow Journal standards as outlined in the editorial comments. Please show fluorochrome axis labels and scaling in flow cytometry plots. Failure to do this will result in delays in the re-review process. We have made these amends and we show fluorochrome axis labels

Reviewer: 1

The abstract does not really provide all essential information about the IgMi mouse model. Not everyone is familiar with this model. Please explain the relevance of this mouse model in more detail. We have now removed the abstract given the Executive committee's recommendation that we convert our manuscript to a "Letter to the editor"

In their manuscript the authors use a mouse model named IgMi and refer to the following reference: Waisman et al., 2007. In Waisman et al., 2007, however, the authors use different nomenclatures for their mouse strains which makes it quite difficult to figure out which mouse they used in the present study. Please be consistent in the description and nomenclature of this mouse line. Waisman et al 2007 used IgHμγ1nomenclature to describe the IgMi mouse. However, subsequently, Waisman adopted the nomenclature IgMi (Waisman et al; 2008 Med Microbiol Immunol 2008, 197 (2): 145-9). We have now included this reference at the start of our Letter (lines 10-12) to clarify the nomenclature and use the IgMi terminology consistently throughout.

I recommend to include a schematic drawing of the genetic features and alterations of the IgMi mouse. Waisman et al 2007, and Waisman et al 2008 have published a schematic; we now refer to both of these references on our Introduction, Lines 10-12.

The authors found enlarged spleens and MLNs in the IgMi mouse. What about transitional B cells. Are they changed?

We have now analysed transitional B cells on the spleen of IgMi and WT littermate controls. We find that this population of B cells is increased. We have now included this data in Figure 1G and commented in the text line 24.

Is B cell development in the bone marrow completely normal? We have now conducted extra experiments analysing B cells in the bone marrow compartment of IgMi and WT C57BL/6 controls and found that pre-B cells and immature B cells were increased whilst mature B cells were decreased in the IgMi mouse. We have added this data to the supporting data (Supplementary Fig.1) and a description in the text at line 28-30.

In Figure 2 the authors present data that show an increase in B1 cells and MZB cells. Are the cell numbers normalized to the increased number of total splenocytes? Our data for B1 cells and MZB cells is presented as total cells and so is normalised to the increased number of total splenocytes. Total cell numbers are shown in Figure 2

What about CD4 and CD8 T cells in the Spleen (and the MLN)? We saw no significant differences in CD4 and CD8 T cells and have added a comment in the text, line 58-59 to mention this as “data not shown”.

Is there more output from the bone marrow? We know that B cell development is altered in the bone marrow of IgMi mice as we have now conducted extra experiments as stated above. Thus we find that pre-B cells and immature B cells were increased whilst mature bone marrow B cells were decreased in the IgMi mouse. We have added this data to supporting data (Supplementary Fig.2) and a description in the text at line 28-30.

Did the authors analyze the B1 compartment in the peritoneum as well? We did analyse B1 cells in the peritoneum. Please see Figure 1E,F

In Figure 4 the authors nicely show an increase in GL7+ GC B cells.
Are IgMi B cells "hyperactive" in general? Is there in vitro data available (LPS Stimulation, anti CD40/IL4 etc..) regarding proliferation, GL7 upregulation, apoptosis? Please clarify this point. We have stimulated IgMi B cells with LPS and these cells certainly make significantly higher levels of IL-10 (Fig 2I,J). Further we also know that the number of GL7+ cells in IgMi are increased after an infection with an intestinal nematode parasite, compared to WT control levels (unpublished). We have now conducted extra experiments analysing proliferation/apoptosis of GC B cells in MLNs. We found that the proliferation of

Ki67+ GC B cells was significantly increased in IgMi mice, whilst the apoptosis Casp-3+ GC B cells was significantly decreased. We have added this data in supplementary Figure 2 and have added a comment in the text, line 50-51.

Based on the finding that IgMi mice harbor more GL7+ GC cells:
What about plasmablasts and plasma cells. Are they generated in this mouse? Is there an enrichment of IgM+ plasmablasts? Plasma cells and plasmablasts are generated in IgMi mice and littermate controls. Levels are very low under the steady state conditions we have been analysing and do not differ between genotype. We have now added a comment to the text line 52-54 to mention this as “data not shown”.

Statistics:
Please clearly state the total number of mice used for every experiment. Sometimes it is not clear; Figure 2: "Data are pooled from two experiments, n=4". Is that n=8 in total? Or was it two times 2 animals resulting in n=4. Please clarify. We apologise that this was not clear; we have amended our text to make it clear that n=8 in total

For n=4 a t-test is not appropriate because of its low statistical power for small n. Please use other appropriate statistical tests. We have reanalysed our data using Mann-whitney test.

Reviewer: 2

Comments to the Author
In this study, Sahputra et al. report the characterization of cell subsets in IgMi mice that cannot secrete antibodies compared to controls. They find alteration in B cell subsets, DC subsets, and TFH cells. Although this is possibly of interest for people who are considering working with these mice, these descriptive results do not reach the level of significance necessary for publication in the European Journal of Immunology. Part of these findings are already known eg B cell subsets abnormalities in the absence of secreted Abs (muS mice or Aid-/-muS mice).

We are disappointed to hear that this reviewer didn’t feel that our results were significant to warrant publication in EJI. We do note that both the other referees concurred that the data was important and useful to other workers in the field. We hope that by converting our manuscript to a Letter to the Editor

the reviewer will now support publication

Comments: Supplementary Figure 3 is missing in the figure files (cited in line 148). Our figures have now changed given the conversion to a Letter to the editor style.

Reviewer: 3

It would be helpful for the authors to emphasize the degree and timing with which WT and IgMi/i mice were housed together. This is particularly important for the microbiota data in Figure 6, but may also be relevant to several other figures.

Moreover, the authors should consider emphasizing the caution with which the microbiota data are discussed. The microbiota literature is becoming clogged with conflicting reports, and at least some of this reflects different approaches to housing and co-housing genotype-disparate mice. Really drilling down on these issues can be quite involved (see PMID 28614717, for example).

We agree that this is a very important point especially in the context of microbiota data. Throughout our studies we have bred our IgMi and WT mice from crossing heterozygote parents therefore mice are littermate controls and have been co-housed from birth. We have re-emphasised our breeding strategy and present caution when discussing our microbiota data (lines 75-78)

Note: typo – Figure 1C is actually 1B. Apologies – figures have now changed with reformatting to Letter to editor style

Second Editorial Decision

21-Sep-2018

Dear Dr. Else,

It is a pleasure to provisionally accept your manuscript entitled "Evaluating the IgMi mouse as a novel tool

to study B cell biology" for publication in the European Journal of Immunology. For final acceptance, please follow the instructions below and return the requested items as soon as possible as we cannot process your manuscript further until all items listed below are dealt with.

Please note that EJI articles are now published online a few days after final acceptance (see Accepted Articles: https://onlinelibrary.wiley.com/toc/15214141/0/ja). The files used for the Accepted Articles are the final files and information supplied by you in Manuscript Central. You should therefore check that all the information (including author names) is correct as changes will NOT be permitted until the proofs stage.

We look forward to hearing from you and thank you for submitting your manuscript to the European Journal of Immunology.

Yours sincerely,
Nadja Bakocevic

on behalf of
Prof. JÃ¼rgen Wienands

Dr. Nadja Bakocevic
Editorial Office
European Journal of Immunology
e-mail: ejied@wiley.com
www.eji-journal.eu
